# Association of Acute-Phase Proteins and IgG with Bovine Respiratory Disease, Seroconversion to Respiratory Infections and Farm-Level Factors in Rearing Calves

**DOI:** 10.3390/ani16040639

**Published:** 2026-02-17

**Authors:** Rohish Kaura, Elisabeth Dorbek-Sundström, Leena Seppä-Lassila, Vera Talvitie, Jarkko Oksanen, Ulla Rikula, Tuomas Herva, Kerli Mõtus, Timo Soveri, Heli Simojoki, Toomas Orro

**Affiliations:** 1 Institute of Veterinary Medicine and Animal Sciences, Estonian University of Life Sciences, Kreutzwaldi 62, 51006 Tartu, Estonia; elisabeth.dorbek-sundstrom@emu.ee (E.D.-S.); kerli.motus@emu.ee (K.M.); toomas.orro@emu.ee (T.O.); 2 Department of Production Animal Medicine, University of Helsinki, Paroninkuja 20, 04920 Saarentaus, Finland; leena.seppa-lassila@fimnet.fi (L.S.-L.); vera.talvitie@ett.fi (V.T.); timo.soveri@helsinki.fi (T.S.); heli.simojoki@helsinki.fi (H.S.); 3 Municipal Veterinarian, Oulu Environment Office, Solistinkatu 2, 90140 Oulu, Finland; kuntoprojekti@gmail.com; 4 Virology Research Unit, Department of Animal Diseases and Food Safety Research, Finnish Food Authority Ruokavirasto, Mustialankatu 3, 00790 Helsinki, Finland; ulla.rikula@fimnet.fi; 5 Atria Ltd., AtriaBeef, Mikkolantie 16, 90310 Oulu, Finland; tuomas.herva@atria.com

**Keywords:** acute-phase proteins, bovine respiratory disease, calf, IgG

## Abstract

This study investigated the associations between acute-phase proteins (APPs) such as serum amyloid A (SAA), haptoglobin (Hp), and albumin (Alb) as well as immunoglobulin G (IgG) with bovine respiratory disease (BRD), seroconversion to respiratory infections and farm-level factors in rearing calves. Datasets were obtained from a randomised trial of 476 calves in Finland that compared morbidity in large- versus small-group housing. Calves were assessed for clinical BRD, and their blood was sampled three times during the first 50 rearing days to measure APPs and IgG concentrations and virus-specific antibodies against *Mycoplasma bovis* (*M. bovis*), bovine respiratory syncytial virus (BRSV), bovine parainfluenza virus 3 (BPIV3), and bovine coronavirus (BCV). Linear mixed-effects regression models showed higher serum SAA and Hp concentrations in calves with clinical BRD. BRSV seroconversion was associated with increased serum SAA and lower Alb while *M. bovis* seroconversion with increased serum Hp. Calves in larger groups had lower serum Hp, SAA and Alb, and pens with higher BRD cases were associated with increased serum SAA and lower Alb. IgG concentration was associated with BRSV seroconversion. These results suggest that early immune monitoring using APPs and IgG could help guide targeted management strategies to improve calf health and welfare.

## Simple Summary

Bovine respiratory disease is a common and costly health issue in young calves, negatively affecting both growth and welfare. This study investigated whether acute-phase proteins (APPs) in blood could serve as biomarkers to guide management strategies aimed at improving health and welfare in calf-rearing systems. We conducted a trial involving 476 calves in Finland over a 50-day period at rearing farms, where the blood of calves was sampled and their health was monitored. We found that calves with signs of respiratory disease, as well as those housed in pens with a higher number of sick animals, had higher levels of APPs in their blood. Interestingly, calves housed in larger pens had lower levels of certain APPs, suggesting that management practices can influence the risk of inflammation-related illness. Additionally, calves with lower levels of immunoglobulin G (IgG) upon arrival at the rearing farms were more likely to become ill early in the rearing period, indicating a protective effect of higher serum IgG at arrival. Our results suggest that early immune monitoring using APPs and IgG may support calf health and welfare, enabling farmers and veterinarians to implement targeted housing and veterinary interventions.

## 1. Introduction

Healthy calves are essential for a successful cattle production system, both in terms of animal welfare and economic outcomes [1]. Poor calf health leads to immediate financial losses and long-term performance setbacks. Europe is a major global producer of beef and veal, with the European Union producing approximately 6.6 million tonnes in 2022, accounting for 15.6% of its total meat production [2]. In Finland, beef production largely depends on raising bull calves that are either pure dairy breeds or crosses between dairy and beef breeds. About two thirds of these calves, usually around 2 to 3 weeks old, are transported from multiple dairy farms first to specialised calf-rearing units. Later, at approximately six months of age, they are moved to specialised beef production farms. The other one third are delivered directly from dairy farms to integrated beef production farms. This transportation practice of calves from multiple farms increases stress levels and infection risks [3].

Calf mortality is multifactorial, resulting from a combination of factors such as infectious agents, maternal causes and substandard housing and management conditions, as well as the commingling of calves from different sources [4]. Among the most significant health challenges in calf rearing are respiratory infections [5], with both upper and lower respiratory tract infections grouped under the umbrella term of bovine respiratory disease (BRD). BRD contributes to calf mortality, increased treatment costs, and long-term declines in animal performance [6]. Studies consistently identify BRD as the primary reason for high treatment rates on calf-rearing farms [3,7]. A recent study in Finland found that 67% of calves in specialised calf-rearing farms received at least one treatment with antibiotics and/or non-steroidal anti-inflammatory drugs (NSAIDs), with 66% of calves receiving antibiotic treatment at least once during the rearing period [7].

Calves rely on passive immunity from dams as their primary defence against diseases. This immunity is transferred in the form of immunoglobulin G (IgG) antibodies found in colostrum, along with leucocytes and immune-modulating factors such as pro-inflammatory cytokines and acute-phase proteins (APPs) [8]. Inadequate transfer of passive immunity to the calves is a major risk factor for increased morbidity and mortality [9]. At around three weeks of age, calves begin producing their own antibodies, marking the onset of active immune development [10]. However, passive immunity presents both benefits and challenges for young calves: while it protects young calves from diseases, it can also interfere with their ability to develop active immunity [11]. During this vulnerable period, around the age of 3 weeks, stressors such as transportation, sorting, and extensive commingling further increase their susceptibility to diseases.

As BRD contributes to calf morbidity and mortality, increased treatment costs (antibiotics and NSAID), and reduced performance, the early detection of respiratory disease through sensitive diagnostic tools could improve calf welfare, support healthy growth, and promote responsible antibiotic use. In calves, APPs like haptoglobin (Hp), serum amyloid A (SAA), and albumin (Alb) are sensitive markers of systemic inflammation, secreted by the liver during the acute-phase response [12]. Previous studies have linked and shown the usefulness of measuring APPs in both naturally occurring and experimental respiratory infections [13,14,15]. A study by Gånheim et al. [16] reported that, when calves were introduced into new farm environments, those originating from herds with a higher disease incidence had higher serum APPs concentrations and lower weight gains than healthier herds. Similarly, the study by Orro et al. [17] found that SAA was a sensitive marker of inflammation, even when clinical signs of respiratory disease were mild to moderate. Since APPs reflect systemic inflammation [15] and disease severity [17] and can help in evaluating overall calf health [16], they may serve as valuable tools for assessing and monitoring diseases, welfare, and general health status in calves from an early age.

Therefore, this study was conducted to investigate changes in serum concentrations of SAA, Hp, Alb, and IgG in calves with clinical signs of BRD, along with their seroconversion status for selected respiratory infections, longitudinally during the rearing period. The data used in this study were collected from calves during farm visits in Finland, as reported by Seppä-Lassila et al. [3], the first publication from this trial. That study investigated the associations between group size and calf health and growth. Here, the dataset from the same trial is used for a different analysis to explore the associations between calf health, immunity, management factors, and inflammatory response.

## 2. Materials and Methods

### 2.1. Study Design and Population

A randomised trial was conducted in a calf-rearing unit in Western Finland from September 2013 to April 2014. Calves were transported to the rearing unit from dairy farms within a 200 km radius. A detailed description of the study design and sampling was provided by Seppä-Lassila et al. [3]. That study reported the associations between group size and calf health and growth, whereas the current study examined the associations between health, immunity, management factors, and inflammatory responses in rearing calves considering group size.

Briefly, this study was conducted in a calf-rearing facility with 18 compartments housing a total of 1440 calves. The calves (mean arrival age: 24.1 ± 9.2 days) were transported from surrounding farms and randomly assigned upon arrival to either a large group (40 calves per pen) or four small groups (10 calves per pen) upon arrival to the facility, using an alternating allocation method. Throughout this study, two specific all-in-all-out calf-rearing compartments were consistently utilised, with 6 batches of approximately 80 calves, each investigated for 50 days of the rearing period, warranting a balanced comparison between group sizes. On average, calves in the small groups came from 7.2 herds per batch (range: 5–9 herds), while those in the large groups originated from 23.8 herds per batch (range: 16–31 herds). The calf batches included the following breeds: Holstein Friesian, Finnish Ayrshire, and dairy–beef breed mix. The majority of calves were male (82.8%), and most of the calves belonged to Holstein Friesian (45.8%) or Finnish Ayrshire (37.4%) breeds as shown in Table 1 and described by Seppä-Lassila et al. [3]. The details of the sample size calculations are given in Seppä-Lassila et al. [3]. Altogether, 476 calves were included in this study, consisting of 238 calves reared in large groups and 238 calves housed in small groups. All calves were housed in an insulated barn, with controlled ventilation, a slatted floor feeding area, a resting area with wood shaving bedding and standard space allocation per calf. Calves had free access to water and acidified milk replacer, consuming an average of 8–9 litres per day. Concentrates and silage were provided ad libitum and no intake was not recorded. It was assumed that all calves were managed according to the current legislation on the source farms, which includes providing adequate colostrum, and feeding with milk or starter feed before transporting to the rearing facility. Further details on animal and sampling-level related variables are provided in Table 1.

### 2.2. Clinical Assessment and Sample Collection

Upon arrival, or the following day, the calves underwent clinical assessments. The following clinical parameters were documented for all calves: rectal temperature (°C), respiratory rate (breaths/min), presence of nasal and ocular discharge (yes/no), auscultation findings, navel or joint swelling, diarrhoea, and demeanour (depression). Farm workers conducted daily observations for clinical signs in the calves from the time of arrival to the end of the 50-day study period. Following the clinical examination, blood samples were collected by veterinary personnel during the first 50 days of the rearing period: first time directly after arrival at the facility or the following day (first sampling time), and then at three-week intervals (second and third sampling times). In total, 476 calves were included at the first sampling time. By the second (n = 471) and third sampling times (n = 469), the number of calves decreased due to mortality or missing data. All in all, 1416 blood samples were collected for laboratory analysis. Calves that showed clinical BRD signs during the 50-day observation period received antimicrobial treatment according to veterinarian’s guidelines. Farm workers administered antimicrobial treatment if calves met at least two of the following criteria for BRD: rectal temperature ≥39.8 °C, visibly rapid breathing (>60 breaths/min), or signs of depression (lethargy, empty stomach, or lying down while others were active). To estimate infection pressure within the pen, the percentage of calves per pen with clinical BRD at the time of each sampling times was calculated (Table 1) and defined as the number of calves diagnosed with BRD divided by the total number of calves present in the pen at that sampling time. Clinical BRD status used in the analyses were based on the diagnoses made by veterinarians through respiratory clinical examinations at the sampling times. Clinical signs potentially attributable to other health conditions (diarrhoea, umbilical swelling, joint inflammation) were recorded as separate variables and mentioned as “other recorded clinical signs” in Table 1. No calves showed depression as an isolated clinical finding.

The primary BRD treatment consisted of a single intramuscular dose of tulathromycin (Draxxin 100 mg/mL, 2.5 mg/kg) and a subcutaneous dose of meloxicam (Metacam 20 mg/mL, 0.5 mg/kg). If additional treatment was required in recurrent BRD cases, oxytetracycline (Terramycin/LA 200 mg/mL, 20 mg/kg) was administered intramuscularly three times every other day, with meloxicam repeated alongside each antibiotic administration. If a calf met only one of the specified clinical BRD criteria, a single subcutaneous dose of meloxicam (Metacam 20 mg/mL, 0.5 mg/kg) was administered. In cases where primary BRD treatment proves ineffective, benzyl penicillin (Ethacilin 300,000 IU/mL or Penovet 300,000 IU/mL, 20,000–30,000 IU/kg) was administered in combination with an NSAID.

### 2.3. Sample Analysis

Blood samples were collected from the jugular veins of calves and were stored on the day of sampling in a Styrofoam box with cooling elements and transported overnight to the laboratory and protected from extreme temperatures. On the following day, serum was separated by centrifugation (2236× *g* for 10 min at room temperature), then frozen and stored at −20 °C until further analysis. Serum SAA concentration was measured using a commercial ELISA kit (Phase SAA kit, Tridelta Ltd., Maynooth, Ireland) following the manufacturer’s instructions for cattle. Initially, all samples were diluted 1:1000, with a maximum standard curve concentration of 150 mg/L. Samples exceeding this concentration were further diluted and re-assayed. The detection limit of the kit was 0.3 mg/L.

Hp concentrations were measured using the haemoglobin assay method by Makimura and Suzuki [18], modified to use tetramethylbenzidine (0.06 mg/mL) as the chromogen [19]. Standard curves for the assay were generated by serial dilution of pooled, lyophilized bovine acute-phase serum. Calibration used a bovine sample with a known Hp concentration, provided by the European Commission Concerted Action Project (QLK5-CT-1999-0153) [20].

Alb concentrations were determined with an automated chemistry analyser (KONE Pro, Thermo Fisher Scientific, Vantaa, Finland), and IgG levels were measured using a commercial ELISA kit (Bio-X Diagnostics, Rochefort, Belgium) following the manufacturer’s instructions. Intra- and inter-assay coefficients of variation (CV%) were <12% for SAA, <11% for Hp, and <16% for IgG.

Virus-specific antibodies were also detected in the blood samples collected at all three sampling times. *Mycoplasma bovis* (*M. bovis*) antibodies were tested using the BIO K302 Monoscreen Ab ELISA (Bio-X Diagnostics). Antibodies against bovine respiratory syncytial virus (BRSV), bovine parainfluenza virus 3 (BPIV3), and bovine coronavirus (BCV) were tested using kits from SVANOVA Biotech (Uppsala, Sweden). Seroconversion to these respiratory pathogens was defined as a two-fold rise in antibody levels, compared to the optical density value determined at the first sampling time. If a calf seroconverted during the study period, the same calf was considered seroconverted at all sampling times (Table 1).

### 2.4. Statistical Analysis

Linear mixed-effects regression models were used to study how clinical respiratory disease, seroconversion to respiratory infections, group size and other farm-level factors were associated with APPs and IgG concentrations in calves during the 50-day rearing period. Four mixed-effects linear regression repeated-measure models were built, with SAA, Hp, Alb, and IgG concentrations as outcome variables.

To achieve normal distributions, SAA and Hp values were logarithmically transformed. Interaction effects were evaluated for all exploratory variables within each sampling time period. Random effects accounted for variability at the batch and pen levels, as well as repeated sampling within the same calf using a first-order autoregressive structure with homogenous variances to account for temporal correlations. As the pen-level random effect was not significant, this level was excluded from all models, and two-level (batch and calf) nested hierarchical models were used instead.

Potential confounders were controlled in the models, including age at first sampling time, breed (Finnish Ayrshire, Holstein Friesian, mixed dairy–beef breed), group size (large group: 40 calves per pen; small group: 10 calves per pen), and sample haemolysis. As serum Hp measurements can be affected by haemolysis, the degree of haemolysis (no haemolysis, moderate haemolysis or severe haemolysis) was evaluated and included in the statistical model for Hp.

The explanatory variables included in the initial models were clinical BRD status at the sampling time, seroconversion to respiratory pathogens (*M. bovis*, BRSV, BPIV3, BCV), days since the last antimicrobial and NSAID treatments, percentage of calves per pen with clinical BRD and presence of other clinical signs such as diarrhoea, umbilical swelling and joint inflammation. Interaction terms with sample time with all predictor variables were included in the model.

A stepwise backward elimination was used to construct the final models, retaining only variables and interactions that were significant at any sampling time. The model results are presented according to each sampling time. Model fit and variance components were evaluated using the intra-class correlation coefficient for batch effects and the correlation between consecutive sampling times within calves. The same model was fitted separately using each sampling time as the reference category, and only statistically significant main effects are presented for each sampling time in the model results tables. The likelihood ratio test was performed for each model to determine the significance of random effects.

Statistical analyses were performed using Stata/IC 14.2 (Stata Corp, College Station, TX, USA), and results were considered statistically significant at *p* ≤ 0.05.

## 3. Results

**Figure 1:**
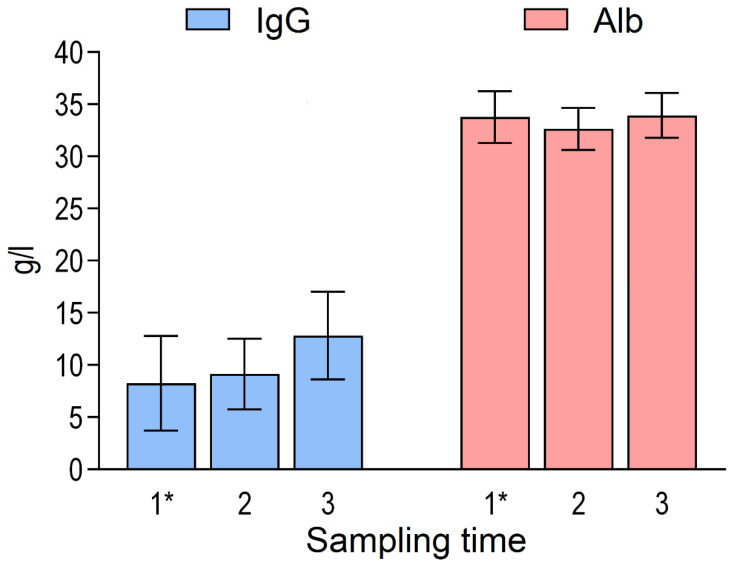
Mean (±SD) serum concentrations of immunoglobulin G (IgG) and albumin (Alb) in calves by sampling time (n = 476, n = 471 and n = 469, respectively). * Results are previously published in Seppä-Lassila et al. [3].

**Figure 2:**
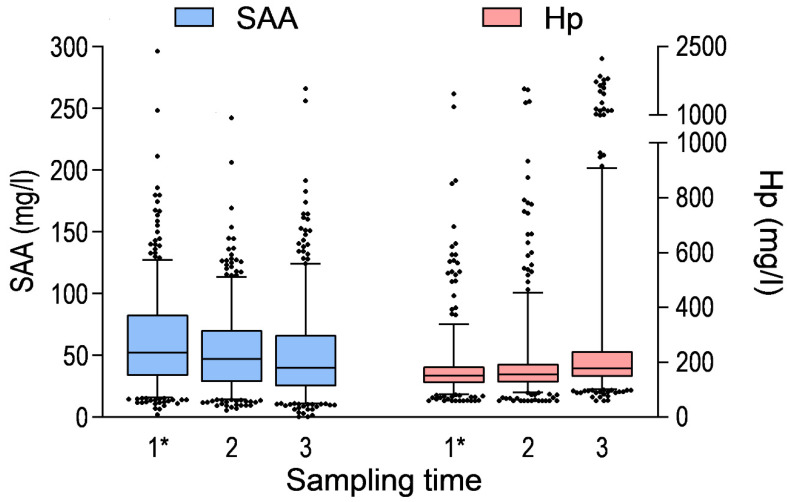
Serum concentrations of serum amyloid A (SAA) and haptoglobin (Hp) in calves by sampling time (n = 476, n = 471 and n = 469, respectively). In this box-and-whisker chart, the box represents the interquartile range and median of the data. The whiskers represent the 5–95% of the data. * Results are previously published in Seppä-Lassila et al. [3].

At the first sampling time, the calves were 25.0 ± 10.2 days old. Clinical signs of disease were present in a proportion of calves at the first sampling time: 5.0% had clinical BRD, 13.4% had diarrhoea, 11.3% showed umbilical swelling, and 0.4% had joint swelling. The descriptive characteristics of animal- and sampling-level variables, as well as concentrations of SAA, Hp, Alb, and IgG, are provided in Table 1 and Figure 1 and Figure 2.

### 3.1. APPs Associations with Clinical BRD

Based on multivariable analysis, calves with clinical BRD had significantly increased serum Hp concentrations at the second (*p* = 0.014) and third sampling time (*p* < 0.001; Table 2). Additionally, calves with clinical BRD had increased serum SAA concentrations at the third sampling time (*p* < 0.001; Table 3). At the first sampling time, a higher percentage of calves per pen with clinical BRD was associated with lower serum Alb (*p* < 0.001; Table 4) and increased serum Hp concentrations (*p* = 0.029; Table 2), whereas at the third sampling time, pens with a higher percentage of calves with clinical BRD had significantly increased serum SAA concentration (*p* = 0.002; Table 3).

### 3.2. APPs and IgG Associations with Seroconversion to Respiratory Infections

Calves that seroconverted to BRSV had significantly lower serum Alb (*p* < 0.001; Table 4) and lower serum IgG concentrations (*p* = 0.015; Table 5) at the first sampling time. Additionally, BRSV seroconversion in calves was significantly associated with increased serum SAA concentrations at the second sampling time (*p* = 0.006; Table 3). At the third sampling time, *M. bovis* seroconversion was significantly associated with increased serum Hp concentration (*p* = 0.035; Table 2). Seroconversion to BPIV3 and BCV in calves were also evaluated; however, no significant associations were found.

### 3.3. Effect of Group Size on APPs Concentrations

Group size had a significant effect on serum APPs concentrations only at the third sampling time. Calves housed in larger groups (40 calves per pen) had lower serum Hp (*p* = 0.011; Table 2), serum SAA (*p* < 0.001; Table 3) and serum Alb concentrations (*p* < 0.001; Table 4) compared to those in smaller groups (10 calves per pen).

### 3.4. Breed and Treatment History Effect on APPs and IgG Concentrations

Holstein calves had lower serum SAA concentrations compared to Finnish Ayrshire calves, with significant differences at the first (*p* < 0.001), second (*p* = 0.019), and third (*p* = 0.022; Table 3) sampling times. Holstein calves also had significantly lower serum IgG concentrations than Finnish Ayrshire calves at the second (*p* = 0.021; Table 5) and third (*p* = 0.004; Table 5) sampling times, while mixed dairy–beef breed calves had lower serum IgG concentrations at the third sampling time compared to Finnish Ayrshire calves (*p* = 0.009; Table 5).

Previous antibiotic and NSAID treatments were significantly associated with APPs and IgG concentrations. Serum SAA concentration was negatively associated with the number of days since the last antibiotic treatment at the second (*p* = 0.036) and third sampling times (*p* = 0.006; Table 3), whereas serum Alb and IgG concentrations were positively associated with days since the last antibiotic treatment at the second (*p* = 0.018; Table 4) and third sampling times (*p* = 0.013; Table 5), respectively. Moreover, days since the last NSAID treatment were positively associated with serum Alb concentration (*p* = 0.022; Table 4) and negatively associated with serum IgG concentration at the third sampling time (*p* = 0.029; Table 5).

## 4. Discussion

### 4.1. Clinical BRD and APPs Responses

The use of APPs as biomarkers in calves is important for understanding their immune status and ability to respond to infections during early life. APPs serve as valuable indicators of inflammation, immune function, and overall health, which are essential factors for effective calf management [14]. With this in mind, the present study investigated APPs serum concentrations in relation to clinical respiratory disease, seroconversion to respiratory infections, group size, and other farm-level factors at three sampling times, over a 50-day period post-arrival in a calf-rearing facility in Finland.

BRD is a leading cause of morbidity and mortality in calves, and Hp is frequently used as a marker of inflammation or infection, especially in respiratory diseases [13,14,21]. The positive associations found in this study between clinical BRD status and serum Hp concentration at the second and third sampling times supports the established role of Hp in responding to respiratory infections. Similarly, the presence of clinical BRD was associated with increased serum SAA concentrations at the third sampling time, which aligns with the role of SAA as an APPs that increases during respiratory infections [15,17,21]. This result also suggests that, at the first sampling time, fewer clinical BRD calves might mean less systemic immune activation in calves. As more calves had developed BRD by the third sampling time, the inflammatory response became more evident, which might explain the delayed increase in APPs response. A higher percentage of calves per pen with clinical BRD was also associated with APPs response, which is reflected in increased serum SAA and lower serum Alb concentrations, agreeing with their roles as positive and negative APPs [22,23].

### 4.2. Associations Between Seroconversion to Respiratory Infections with APPs and IgG

Calves that seroconverted for BRSV had significantly lower serum Alb concentrations at the first sampling time and increased serum SAA at the second sampling time. BRSV infections can elicit widespread inflammation, causing acute-phase response and further decrease in serum Alb [23] and increase in serum SAA concentrations as part of the inflammatory process [17].

Moreover, the associations between *M. bovis* seroconversion and increased serum Hp concentration in calves at the third sampling time suggests that calves exposed to *M. bovis* also have increased inflammatory responses. As a well-known pathogen associated with respiratory disease, *M. bovis* exposure is linked to chronic inflammation, specifically if co-pathogens worsen disease outcomes in *M. bovis*-infected calves [14,24]. This also explains why there was a delayed increase in serum Hp: it is likely due to the subclinical nature of *M. bovis* [25,26], which may not cause an acute-phase response after exposure or may cause a response only in cases with co-infections.

We also found that serum IgG concentrations were significantly lower in calves that had seroconverted to BRSV at the first sampling time, suggesting that infections like BRSV may impair IgG production [27]. Alternatively, it is also possible that calves with lower IgG were more vulnerable to earlier BRSV infection. Maternal antibodies play a protective role and can reduce the severity of clinical disease [28]; however, this observation can also be due to the presence of maternal antibodies in the case of BRSV [29], which delays the calf’s immune response and hinders its ability to develop a strong defence. In our study design, IgG was measured only at arrival, and we lacked information on colostrum management, timing of BRSV exposure, or other pre-arrival management factors. Therefore, we cannot distinguish what causes the observed lower IgG concentrations from pre-arrival factors, and IgG measured at arrival should be interpreted primarily as a proxy marker of early-life immune protection and management. Nevertheless, this highlights the complex but important role of IgG in immune protection and disease resilience in the face of common respiratory infections during the early calf-rearing period.

### 4.3. Effect of Housing on Calf Serum APPs Concentrations

At the third sampling time, calves in larger groups had lower serum SAA, Hp and Alb concentrations. This finding is intriguing, as it conflicts with the common understanding that housing calves in larger groups increases the risk of infection [30], and consequently the inflammatory response [31], compared to smaller groups that may present a lower infection pressure. Svensson and Liberg [30] found no significant differences in mean Hp concentrations between calves kept in the small-sized (6–9) versus the large-sized groups (12–18). In our models, we accounted for pen-level infection pressure at each sampling time and calf’s clinical BRD status. This suggests that the higher APPs concentrations observed in smaller groups was probably not explained completely by differences in disease burden.

Results from behavioural and physiological studies indicates that social housing can affect calf stress responses and immune signalling in complex ways. For example, ref. [32] reported that calves with previous group-housing experience showed a reduced regrouping stress response than individually housed calves, including lower heart rate and salivary cortisol responses and positive behavioural profiles after regrouping. Lv et al. [33] found that group-housed calves expressed more social and exploratory behaviours than individually housed calves, and also reported higher cytokine concentrations (interleukin-2, interleukin-6, tumour necrosis factor-α) in grouped calves. These studies indicate that group size can affect immunity, behaviour and physiological stress. In our study, the lower APPs concentrations in larger groups could reflect differences in inflammatory response during the rearing period; however, because we did not measure behaviour, cortisol, or autonomic indicators, our interpretations are speculative.

Alb is a negative APPs and suggests inflammatory processes; however, it is different from the other APPs and can respond to multiple physiological processes such as metabolic and nutritional demands or other non-inflammatory conditions [34]. Therefore, Alb should be interpreted cautiously in this context.

### 4.4. Other Influential Factors Affecting APPs and IgG Concentrations

In our study, serum SAA concentrations in calves varied between breeds: Holstein calves had lower serum SAA compared to Finnish Ayrshire calves at the first, second and third sampling times, while mixed dairy–beef breed calves showed no significant difference compared to Finnish Ayrshire, suggesting breed-specific differences in immune response [35]. This difference may be influenced by Holsteins’ genetic selection for high milk production, which may influence their immune responses differently compared to breeds like Finnish Ayrshire [36]. Breed was also associated with serum IgG concentrations, with Holstein calves showing significantly lower serum IgG than Finnish Ayrshire calves at both the second and third sampling times and mixed dairy–beef breed calves only at the third sampling time. This aligns with research suggesting that genetic differences affect immune resilience, with some breeds being more resistant to infections [37]. Additionally, the Finnish Ayrshire breed is more common in traditional, smaller herds, whereas Holsteins are predominantly reared in larger, more intensive production systems. It is reported that a larger herd size is a significant risk factor for inadequate colostrum management [38].

The negative associations between days since the last antibiotic treatment and serum SAA concentration suggests that antibiotics, by reducing infection and inflammation [39], significantly lower SAA serum concentration in calves. Moreover, the positive associations between serum Alb concentration and days since the last antibiotic and NSAID treatment shows recovery from inflammation where the liver resumes normal albumin production [40]. Together, these associations may suggest the potential use of APPs to monitor treatment effectiveness. Days since last antibiotic treatments showed a significant positive relationship with serum IgG concentrations, indicating active IgG production during the recovery phase post-infection. Furthermore, a negative association between days since last NSAID treatment and serum IgG concentration was also observed, which may be due to NSAID anti-inflammatory effects that can suppress immune responses [41,42].

### 4.5. Limitations

The nature of the observational part of this study limits making causal inferences due to the potential for residual confounding from unmeasured pen-level factors such as ventilation efficacy, transport stress, etc. While the confounding effect of breed was controlled in models, it may still act as a proxy for underlying management and environmental differences, since calves originated from different herds. Another limitation is that, while seroconversion indicates whether calves were exposed to respiratory pathogens and developed an immune response, we lack the information about the exact time of infection. As a result, some infection-related effects on APPs responses may not have been fully accounted for in our analyses. Although the models controlled for several confounders, unmeasured factors at the pen or herd level could still influence the outcomes. Therefore, the findings must be interpreted in light of potential field-level variations, as some associations may be influenced by confounding factors or by interactions not captured in the models.

## 5. Conclusions

This study found that calves with clinical BRD and those that seroconverted to BRSV and *M. bovis* showed changes in serum APPs concentration, indicating an acute-phase response to pathogen exposure. Additionally, calves housed in larger pens had lower serum Hp, SAA and Alb concentrations, highlighting the influence of management practices on calves’ probability of suffering from inflammation-related diseases. Calves with lower serum IgG concentration at arrival to the facility were more likely to seroconvert to BRSV, suggesting an important complex role of IgG in immune protection and disease resilience during the early calf-rearing period. While more research is needed to assess APPs feasibility for routine farm use, our results highlight their potential value in calf health and welfare monitoring.

## Figures and Tables

**Table 1:** Descriptive characteristics of animal- and sampling-level variables.

Variable		Mean (±SD)/n (%)	
Age for calves at first sampling time (days)		25.0 ± 10.2	
**Sex:**			
Male	394 (82.8%)		
Female	82 (17.2%)		
**Breed:**			
Finnish Ayrshire	178 (37.4%)		
Holstein Friesian	218 (45.8%)		
Mixed dairy–beef breed	80 (16.8%)		
**Calf seroconversion ^1^** **to respiratory pathogens:**			
*Mycoplasma bovis* (n, %)	8 (1.7%)		
BRSV ^2^ (n, %)	134 (28.6%)		
BCV ^3^ (n, %)	106 (22.3%)		
BPIV3 ^4^ (n, %)	79 (16.6%)		
**Bovine respiratory disease (BRD) and treatment**	**First sampling time (n = 476)**	**Second sampling time (n = 471)**	**Third sampling time (n = 469)**
Number of pens with ≥1 calf with clinical BRD	30	30	30
Mean % of calves per pen with clinical BRD	4.8%	11.2%	8.6%
Calves with clinical BRD (n, %)	24 (5.0%)	45 (9.6%)	50 (10.7%)
Median days since last antimicrobial treatment (min–max)	0 (0–1)	8 (1–22)	16 (1–43)
Median days since last NSAID ^5^ treatment (min–max)	0 (0–1)	11 (1–22)	20 (1–43)
**Other recorded clinical signs**			
Diarrhoea (n, %)	64 (13.4%)	108 (23.0%)	25 (5.3%)
Umbilical swelling (n, %)	54 (11.3%)	24 (5.1%)	27 (5.8%)
Swelling of joints (n, %)	3 (0.4%)	1 (0.2%)	4 (0.9%)

^1^ Seroconversion was defined as a two-fold rise in antibody levels between consecutive samplings. If a calf was seroconverted during the study period, the same calf was considered seroconverted at all sampling times (first, second and third). ^2^ BRSV = bovine respiratory syncytial virus. ^3^ BCV = bovine coronavirus. ^4^ BPIV3 = bovine parainfluenza virus 3. ^5^ NSAID = non-steroidal anti-inflammatory drugs.

**Table 2:** Results of multivariable linear mixed-effects regression model analysing the associations of haptoglobin (Hp) concentration (log (mg/L)) with clinical respiratory disease, seroconversion to respiratory infections, group size, and other farm-level factors in calves during the 50-day rearing period by sampling time: at the beginning (n = 476), in the middle (n = 471), and at the end (n = 469). The batch (n = 6) and sample time of the calf with first-order autoregressive structure with homogenous variances (AR1) were included as random factors. The same model was fitted separately with each sampling time as the reference category, and only significant main effects are presented. These main effects represent associations at the indicated sampling times.

Variable	n	Coeff.	SE	*p*-Value	Wald Test *p*-Value
**At first sampling time**					
Percentage of calves per pen with clinical BRD ^1^	476	0.009	0.004	0.029	
Intercept		5.043	0.083	<0.001	
**At second sampling time**					
Breed:					0.056
Finnish Ayrshire	176				
Holstein Friesian	215	−0.116	0.053	0.030	
Mixed ^2^	80	−0.131	0.071	0.065	
Clinical BRD ^1^:					
No	426	0			
Yes	45	0.202	0.082	0.014	
*M. bovis* ^3^ seroconversion:					
No	464	0			
Yes	7	−0.351	0.193	0.070	
Intercept		5.274	0.105	<0.001	
**At third sampling time**					
Breed:					0.071
Finnish Ayrshire	175				
Holstein Friesian	214	−0.139	0.069	0.043	
Mixed ^2^	80	−0.092	0.052	0.077	
Group size:					
Small (10 calves per pen)	236	0			
Large (40 calves per pen)	233	−0.120	0.048	0.011	
Clinical BRD ^1^:					
No	419	0			
Yes	50	0.619	0.076	<0.001	
Days since last antibiotic treatment	469	−0.004	0.002	0.062	
*M. bovis* ^3^ seroconversion:					
No	461	0			
Yes	8	0.384	0.182	0.035	
Intercept		5.471	0.066	<0.001	

Intra-class correlations coefficient (ICC) for batch = 0.025 and correlation between two following sampling times rho = 0.132. Likelihood ratio test *p* < 0.001 for random effect. ^1^ Bovine respiratory disease. ^2^ Dairy and beef cattle mixed breed. ^3^ *Mycoplasma bovis*.

**Table 3:** Results of multivariable linear mixed-effects regression model analysing the associations of serum amyloid A (SAA) concentration (log (mg/L)) with clinical respiratory disease, seroconversion to respiratory infections, group size, and other farm-level factors in calves during the 50-day rearing period by sampling time: at the beginning (n = 476), in the middle (n = 471), and at the end (n = 469). The batch (n = 6) and sample time of the calf with first-order autoregressive structure with homogenous variances (AR1) were included as random factors. The same model was fitted separately with each sampling time as the reference category, and only significant main effects are presented. These main effects represent associations at the indicated sampling times.

Variable	n	Coeff.	SE	*p*-Value	Wald Test *p*-Value
**At first sampling time**					
Age at first sampling time (day)	476	−0.052	0.012	<0.001	
Age at first sampling time ^squared^ (day)	476	0.0005	0.0002	0.014	
Breed:					<0.001 *
Finnish Ayrshire	178	0			
Holstein Friesian	218	−0.257	0.067	<0.001	
Mixed ^1^	80	−0.046	0.091	0.610	
Intercept		4.891	0.206	<0.001	
**At second sampling time**					
Age at first sampling time (day)	471	−0.008	0.003	0.022	
Breed:					0.058
Finnish Ayrshire	176	0			
Holstein Friesian	215	−0.157	0.067	0.019	
Mixed ^1^	80	−0.050	0.067	0.572	
Days since last antibiotic treatment	471	−0.007	0.004	0.036	
BRSV ^2^ seroconversion:					
No	338	0			
Yes	133	0.193	0.070	0.006	
Intercept		4.024	0.129	<0.001	
**At third sampling time**					
Age at first sampling time (day)	469	−0.013	0.003	<0.001	
Breed:					0.060
Finnish Ayrshire	175	0			
Holstein Friesian	214	−0.154	0.067	0.022	
Mixed ^1^	80	−0.135	0.090	0.133	
Group size:					
Small (10 calves per pen)	236	0			
Large (40 calves per pen)	233	−0.326	0.070	<0.001	
Clinical BRD ^3^:					
No	419	0			
Yes	50	0.379	0.102	<0.001	
Days since last antibiotic treatment	469	−0.008	0.003	0.006	
Percentage of calves per pen with clinical BRD ^3^	469	0.013	0.004	0.002	
*M. bovis* ^4^ seroconversion:					
No	461	0			
Yes	8	0.429	0.234	0.068	
Intercept		4.183	0.121	<0.001	

Intra-class correlations coefficient (ICC) for batch = 0.022 and correlation between two following sampling times rho = 0.055. Likelihood ratio test *p* < 0.001 for random effect. ^1^ Dairy and beef cattle mixed breed. ^2^ Bovine respiratory syncytial virus. ^3^ Bovine respiratory disease. ^4^ *Mycoplasma bovis*. * Results are previously published in Seppä-Lassila et al. [3].

**Table 4:** Results of multivariable linear mixed-effects regression model analysing the associations of albumin (Alb) concentration (g/L) with clinical respiratory disease, seroconversion to respiratory infections, group size, and other farm-level factors in calves during the 50-day rearing period by sampling time: at the beginning (n = 476), in the middle (n = 471), and at the end (n = 469). The batch (n = 6) and sample time of the calf with first-order autoregressive structure with homogenous variances (AR1) were included as random factors. The same model was fitted separately with each sampling time as the reference category, and only significant main effects are presented. These main effects represent associations at the indicated sampling times.

Variables	n	Coeff.	SE	*p*-Value
**At first sampling time**				
Age at first sampling time (day)	476	0.222	0.039	<0.001
Age at first sampling time ^squared^ (day)	476	−0.003	0.001	<0.001
Percentage of calves per pen with clinical BRD ^1^	476	−0.098	0.015	<0.001
BRSV ^2^ seroconversion:				
No	342	0		
Yes	134	−1.057	0.223	<0.001
Intercept		31.068	0.620	<0.001
**At second sampling time**				
Age at first sampling time (day)	471	0.038	0.011	<0.001
Days since last antibiotic treatment	471	0.030	0.013	0.018
Intercept		31.210	0.426	<0.001
**At third sampling time**				
Age at first sampling time (day)	469	0.046	0.011	<0.001
Days since last NSAID ^3^ treatment	469	0.009	0.003	0.022
Group size:				
Small (10 calves per pen)	236	0		
Large (40 calves per pen)	233	−1.119	0.256	<0.001
Intercept		32.889	0.408	<0.001

Intra-class correlations coefficient (ICC) for batch = 0.047 and correlation between two following sampling times rho = 0.385. Likelihood ratio test *p* < 0.001 for random effect. ^1^ Bovine respiratory disease. ^2^ Bovine respiratory syncytial virus. ^3^ Non-steroidal anti-inflammatory drugs.

**Table 5:** Results of multivariable linear mixed-effects regression model analysing the associations of immunoglobulin G (IgG) concentration (g/L) with clinical respiratory disease, seroconversion to respiratory infections, group size, and other farm-level factors in calves during the 50-day rearing period by sampling time: at the beginning (n = 476), in the middle (n = 471), and at the end (n = 469) of 50 days of rearing period. The batch (n = 6) and sample time of the calf with first-order autoregressive structure with homogenous variances (AR1) were included as random factors. The same model was fitted separately with each sampling time as the reference category, and only significant main effects are presented. These main effects represent associations at the indicated sampling times.

Variables	n	Coeff.	SE	*p*-Value	Wald Test *p*-Value
**At first sampling time**					
Age at first sampling time (day)	476	0.141	0.02	<0.001	
BRSV ^1^ seroconversion:					
No	342	0			
Yes	134	−1.054	0.431	0.015	
*M. bovis* ^2^ seroconversion:					
No	468	0			
Yes	8	−2.389	1.416	0.092	
Intercept		5.549	0.693	<0.001	
**At second sampling time**					
Age at first sampling time (day)	471	0.139	0.02	<0.001	
Breed:					0.043
Finnish Ayrshire	215	0			
Holstein Friesian	176	−0.925	0.402	0.021	
Mixed ^3^	80	−0.062	0.537	0.939	
Intercept		6.931	0.741	<0.001	
**At third sampling time**					
Age at first sampling time (day)	469	0.099	0.020	<0.001	
Breed:					0.005
Finnish Ayrshire	214				
Holstein Friesian	175	−1.164	0.403	0.004	
Mixed ^3^	80	−1.406	0.541	0.009	
Days since last antibiotic treatment	469	0.033	0.013	0.013	
Days since last NSAID ^4^ treatment	469	−0.013	0.006	0.029	
Intercept		10.728	0.730	<0.001	

Intra-class correlations coefficient (ICC) for batch = 0.029 and correlation between two following sampling times rho = 0.587. Likelihood ratio test *p* < 0.001 for random effect. ^1^ Bovine respiratory syncytial virus. ^2^ *Mycoplasma bovis*. ^3^ Dairy and beef cattle mixed breed. ^4^ Non-steroidal anti-inflammatory drugs.

## Data Availability

The data is contained within the article.
